# Failure Analysis of the Tree Column Structures Type AlSi10Mg Alloy Branches Manufactured by Selective Laser Melting

**DOI:** 10.3390/ma13183969

**Published:** 2020-09-08

**Authors:** Peikang Bai, Pengcheng Huo, Taotao Kang, Zhanyong Zhao, Wenbo Du, Minjie Liang, Yuxin Li, Haihong Liao, Yun Liu

**Affiliations:** 1 School of Materials Science and Engineering, North University of China, Taiyuan 030051, China; baipeikang@nuc.edu.cn (P.B.); nuchpc@126.com (P.H.); l18734159668@163.com (M.L.); liyuxin@nuc.edu.cn (Y.L.); lhh@nuc.edu.cn (H.L.); 2 Capital Aerospace Machinery Corporation Limited, Beijing 100072, China; zhenshilu@126.com; 3 National Key Laboratory for Remanufacturing, Army Academy of Armored Forces, Beijing 100072, China; dwbneu@163.com

**Keywords:** AlSi10Mg alloy branches, stress, fracture, dimples, Si content distribution, selective laser melting (SLM)

## Abstract

AlSi10Mg alloy branches were fabricated by selective laser melting (SLM), and the branches were employed to evaluate their effect on the mechanical properties. When the porous branches were compressed along its building direction, the tree column structures-type AlSi10Mg alloy branches collapsed twice, which had typical elastic, shear, collapse, and densification stages. The compressive stress concentration at the interface between the support and the porous body caused the fracture of the tree column structures-type AlSi10Mg alloy branches. The fracture surface indicated that the prepared tree-type branches were distributed with different shapes of dimples, and the Si content inside the dimples was higher than that of the edge. The morphology of the Al-Si eutectic structure formed by SLM and the stress concentration at the Al/Al-Si-eutectic interface affected the fracture morphology and Si content distribution.

## 1. Introduction

The AlSi10Mg alloy materials are widely used in the building industry (especially for bridges) due to its good lightweight, high specific strength, and stable coefficient of thermal expansion [1,2,3,4,5,6,7]. For example, the density ranges between 2.70 and 2.83 g/cm^3^, the compressive strength is between 200 and 550 MPa, and the elastic modulus ranges from 69 to 76 GPa [8,9]. Moreover, the compressive strength of AlSi10Mg far exceeds the compressive strength required in the building field, which provides the justification for further research on the lightweight of AlSi10Mg alloy [10].

The controlled porous structure design can address these concerns—for example, three-dimensional periodic minimal surface scaffolds (TPMS) [11,12], honeycomb scaffolds [13,14], and foam scaffolds [15,16]. They have obvious advantages: they are lightweight and less consumable, and they also meet the mechanical properties required by the building field by adjusting the wall thickness, pore shape, and pore distribution of the porous structure. However, at present, the manufacturing of alloy components used in the building field still mainly relies on traditional processing techniques such as casting, machining, forging, and welding. Furthermore, as for the processing of the porous structure, the production cycle of these processes is relatively long; particularly, for some complex precision alloy components, it is almost impossible to use the above-mentioned traditional technology for precise forming.

Recently, SLM is the most potential manufacturing process in additive manufacturing technology [17,18,19]. Simultaneously, compared with other forming methods (such as casting, forging, welding, and machining), SLM breaks through the limitation of the existing process for complex parts, which can realize the integration of structure, manufacturing, and function [20,21]. For example, Leary et al. analyzed the mechanical strength of AlSi10Mg block support structures prepared by SLM and showed that the peel strength of the support structure was significantly less than its tensile strength [22]. Ataee et al. studied the nanoindentation behavior, wear resistance, and in vitro biocompatibility of commercial pure Ti (CP-Ti) prepared by SLM and Ti64 scaffolds prepared by the Electron Beam Melting (EBM) compared to those of casting CP-Ti. It was found that for the CP-Ti made by SLM and the Ti64 made by EBM, the lateral wear resistance is higher than that in the building direction, up to approximately 25% and 82%, respectively. In the nanoindentation test, the wear resistance of CP-Ti stents made by SLM was significantly improved compared to the densely cast CP-Ti, and the average wear height was reduced by 75%. In in vitro cell culture studies, CP-Ti scaffolds prepared by SLM showed higher cell viability and cell adhesion density at all unit cell sizes compared to Ti64 scaffolds [23]. Additionally, based on the characteristics of the porous scaffolds, the shapes of tree branches (complex and fractal-like) also have a series of mechanical and biological functions. The relation between them always draws the attention of architects focusing on the shape and structural strength [24]. For instance, Zhu et al. found that tree-shaped structure is an effective design method for a lightweight support structure in the SLM process, and they proposed an improved particle swarm optimization (PSO) strategy combining the experimental method and volume minimization framework. The results showed that this approach can effectively reduce the volume of support and printing time [25]. Zhang et al. fabricated two types of support structures (branch and lattice) by SLM. The analysis and experiment showed that the new design of the support structures had great potential in getting higher efficiency and reducing the manufacturing cost (for example, when the diameter of support is 1.5 mm and the inclination angle of the branch is 45 degrees, the lightweight ratio is 23% in practice and the theoretical value is 39% compared with the opposite lattice support) [26].

Thus far, the structural design of porous alloy branches is mostly limited to TPMS, honeycomb, and foam branches. There are few publications about the tree column structures-type alloy branches combined with a dense body and porous body. Moreover, porous branches with different structures show different mechanical properties. Therefore, this study is different from the traditional porous alloy branches; design inspired from underwater lightweight tree column structures was considered. These specific structural designs have shapes such as voids or bubble foam, into which air masses get entrapped and maintain stability. Therefore, for the tree column structures type, alloy branches have been designed and manufactured, and the mechanical behavior and fracture mechanism of the porous branches were investigated through finite element methods (FEM) and compression tests.

## 2. Experimental Procedure

### 2.1. Materials and SLM process

**Figure 1:**
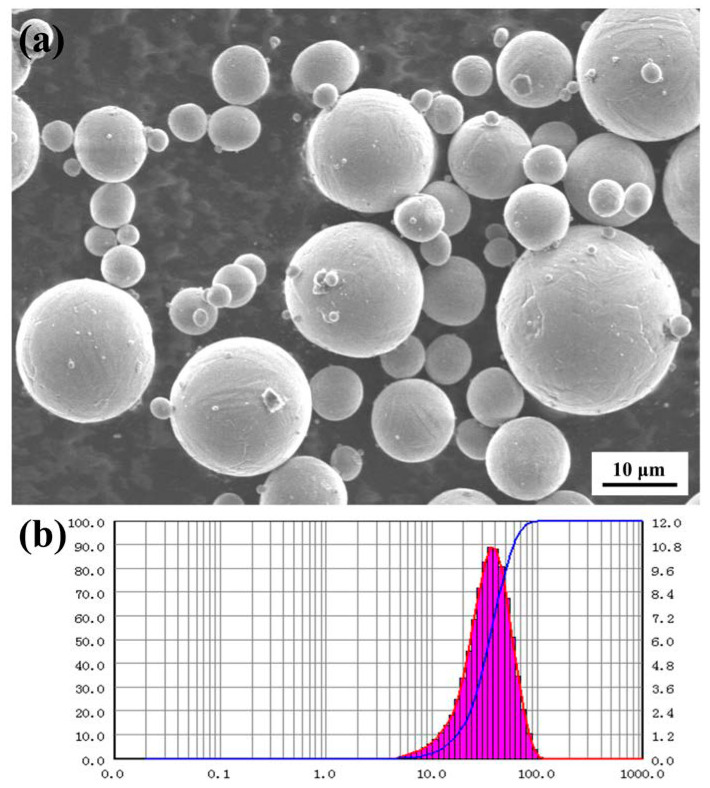
Spherical alloy powder and particle size distribution used in the experiments: (**a**) SEM image of AlSi10Mg powder; (**b**) particle size distribution of AlSi10Mg powder.

Spherical alloy powder (Beijing e-Plus 3D Tech. Co., Ltd., Beijing, China) with the following main compositions: 89.35 wt % Al, 9.8 wt % Si and 0.35 wt % Mg (Table 1); particle sizes: D10 = 17.63 µm, D50 = 35.33 µm, and D90 = 59.91 µm. The SEM micrograph of AlSi10Mg powders and the particle size distribution are shown in Figure 1. All alloy branches were prepared by Renishaw AM 400, including a Yb fiber laser (wavelength 1070 nm). During the SLM process, the scanning speed was 1300 mm/s, the laser power was 370 W, and the exposure time (ET) was 150 µs. A chessboard scanning strategy was adopted with a rotation of 67° in each layer prior to the next exposure (The scanning strategy (67° rotation) caused a randomly oriented and finer grain structure with a higher ultimate tensile strength (UTS) [27]). The SLM process was under the protection of argon, and the oxygen content was less than 0.05%. The substrate was preheated to 180 ℃ before SLM processing.

### 2.2. Finite Element Modeling

**Figure 2:**
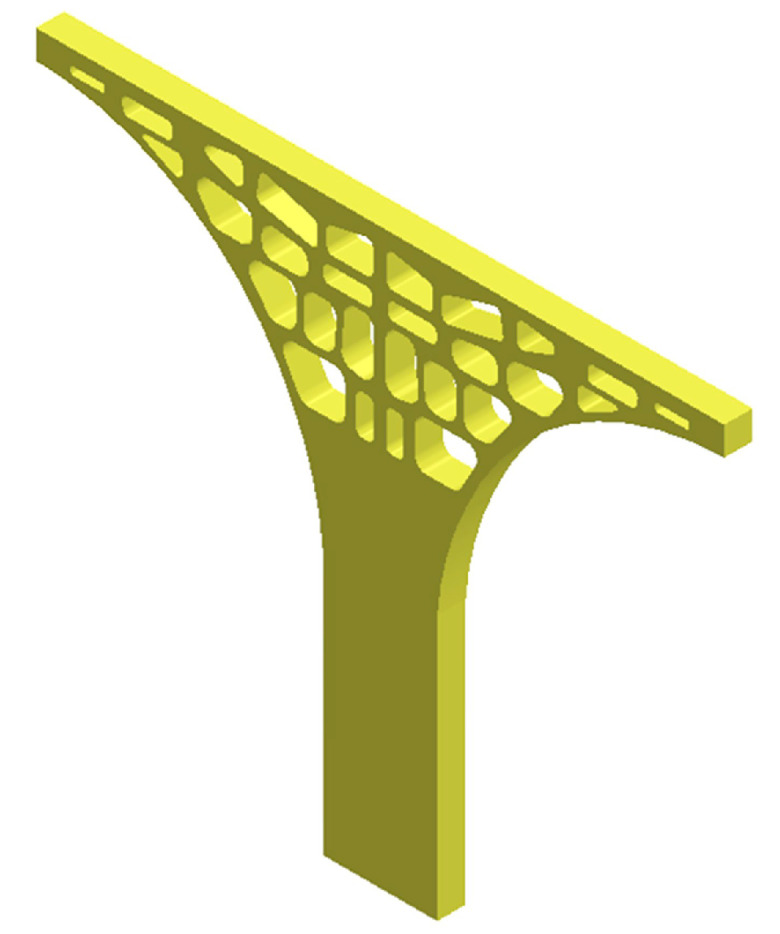
Three-dimensional computer-aided design (3D-CAD) model of the tree column structure.

**Figure 3:**
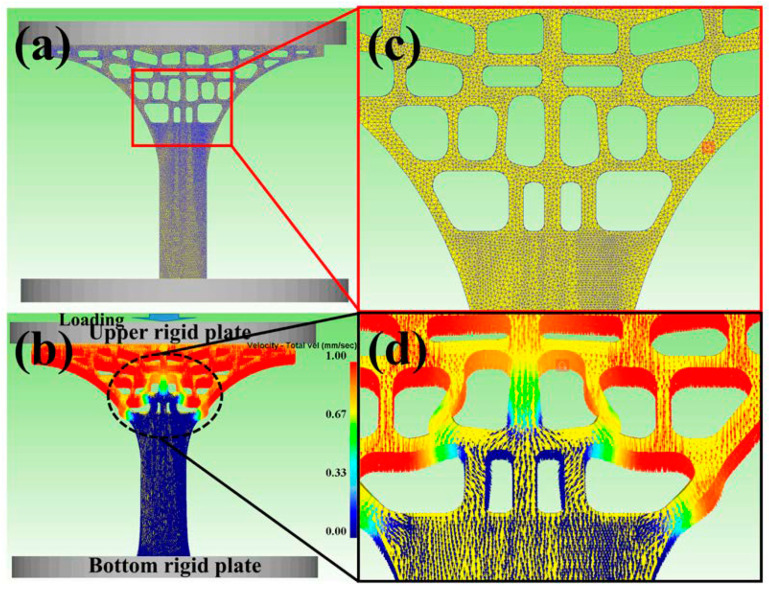
(**a**) The 3D-CAD model meshed with tetrahedral elements; (**c**) the enlarged view showing the FEM meshes; (**b**) The loading diagram by the speed loading; (**d**) the magnified view showing the velocity vector.

A three-dimensional computer-aided design (3D-CAD) model of the tree column structure was established in 3D design software SolidWorks with a thickness of 5 mm (Figure 2). A 3D-CAD model was meshed with tetrahedral elements (Figure 3a,c), and the size of the elements was automatically allocated by the Deform-3D finite element simulation software in an optimized way (the number of elements is 456,450). Constraints and loading conditions were carried out based on the actual compression environment (bottom rigid plate and upper rigid plate, Figure 3b). The bottom rigid plate was fixed, whilst the upper rigid plate was subjected to a prescribed velocity as loading conditions (1 mm/s, Figure 3b,d). Based on the inherent material properties of AlSi10Mg, an elastoplastic constant was defined for the 3D-CAD model with a Young’s modulus of 69 GPa and Poisson’s ratio of 0.33.

### 2.3. Microstructural Observation and Mechanical Tests

The tree column structures-type AlSi10Mg alloy branches were mechanically polished via SiC sandpaper and etched with 95 mL H_2_O + 2.5 mL HNO_3_ + 1.5 mL HCl + 1 mL HF for 15 s. The microstructure of tree column structures-type AlSi10Mg alloy branches were performed by optical microscopy (OM, Axiovert 200 MAT, Zeiss, Munich, Germany) and SEM (JSM-7900F, JEOL Ltd., Fukuoka, Japan) equipped an energy-dispersive spectroscopy (EDS). For phase analysis, X-ray diffraction (XRD) was performed by a D8 ADVANCE A25 machine (Bruker AXS, Karlsruhe, Germany). Uniaxial compression tests (the compression speed was 1 mm/s) were carried out at room temperature using a SANS-CMT5105 testing machine (Shenzhen SANS Testing Machine Company, Ltd., Shenzhen, China). At least three AlSi10Mg alloy branches were used to ensure the precision and reliability of the data. The deformation of the AlSi10Mg alloy branches was recorded in a Single Lens Reflex (SLR) digital camera (Nikon Imaging China Sales Co., Ltd., Beijing, China).

## 3. Results and Discussions

**Figure 4:**
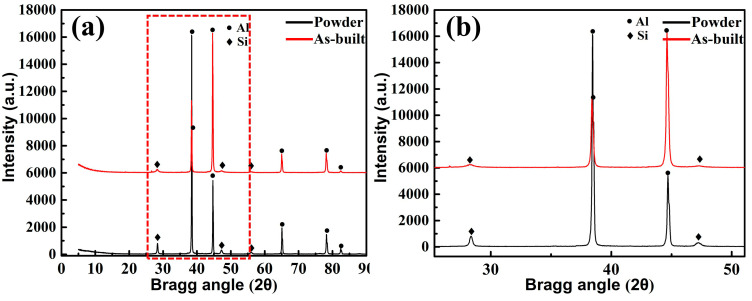
XRD patterns of AlSi10Mg powder and as-built AlSi10Mg alloy branch: (**a**) Complete XRD pattern, (**b**) Magnified XRD pattern of the local area.

To identify the phase change between porous alloy branches in SLM process and AlSi10Mg powder, XRD patterns were carried to them. Figure 4 presents the XRD spectra of the AlSi10Mg powder and the as-built AlSi10Mg alloy branch. The XRD patterns mainly consisted of *α*-Al and a eutectic Si phase. The lower peak value of Si phase compared with the Al phase is mainly due to the lower Si content of raw material powder. Moreover, the content of Mg in the powder is low, and the formation of Mg_2_Si in the alloy is hardly revealed. Particularly, for the Si phase, it can be found that the peak intensity of the Si phase of the built alloy scaffold is weaker than that of the powder sample (Figure 4b). The unique rapid melting and solidification of SLM make the kinetics to form large eutectic structures unfavorable, so the Si tends to remain in solution with the Al primary matrix.

**Figure 5:**
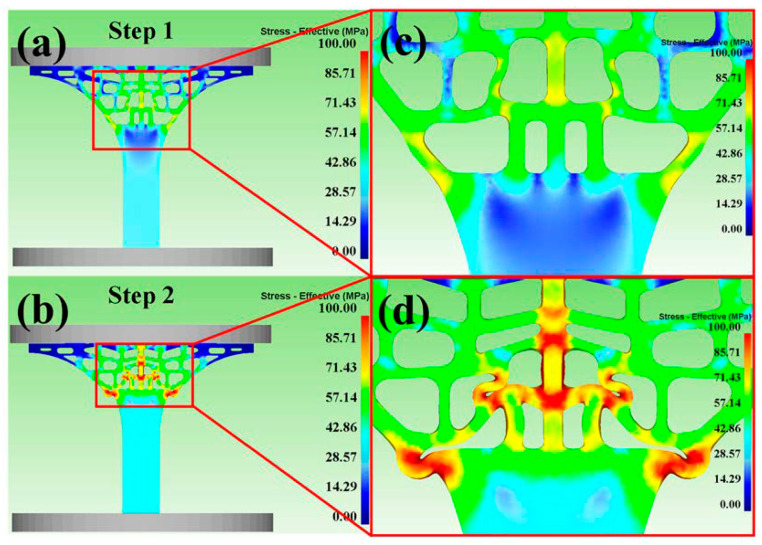
Predicted effective stress distribution during compression at the different steps: (**a**,**b**) Stress-effective at Step 1 and Step 2; (**c**,**d**) the enlarged view.

**Figure 6:**
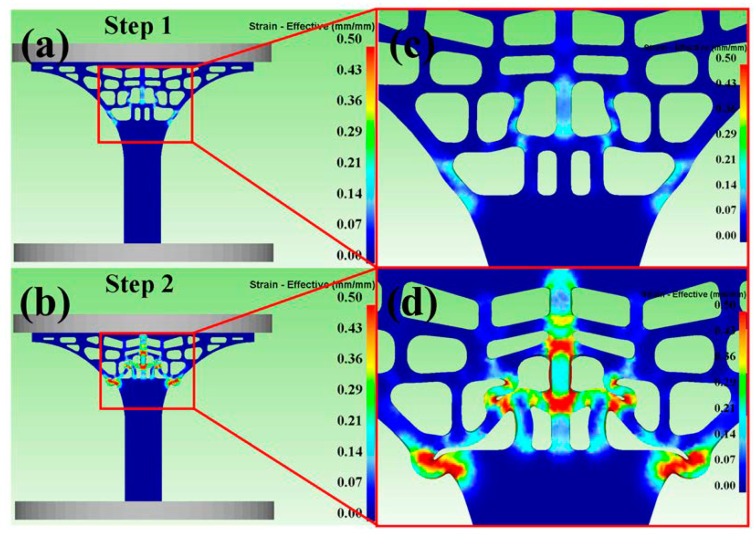
Predicted effective-strain distribution during compression at the different steps (**a**,**b**) Strain-Effective at Step 1 and Step 2; (**c**,**d**) the magnified view.

Figure 5 and Figure 6 showed the distribution of stress and strain of the tree column structures-type AlSi10Mg alloy 3D CAD model at Step 1 and Step 2 (Step 1 and Step 2 present that the upper rigid plate has been running for 1s, 5s). When the 3D-CAD model was in the initial stage of compression (Step 1), the stress-strain distribution of the scaffold was relatively uniform, and only slight deformation occurred (Figure 5a,c) and (Figure 6a,c). The compression response indicated that the stress-strain concentration position of the tree column structures-type AlSi10Mg alloy branches designed in this study was the weak position of mechanical properties. After reaching for Step 2, there were large deformations and bending in the weak position of the 3D-CAD model (Figure 5b,d) and (Figure 6b,d). Therefore, a key conclusion is that the weak position of the tree column structures-type AlSi10Mg alloy branches was bent and fractured preferentially.

**Figure 7:**
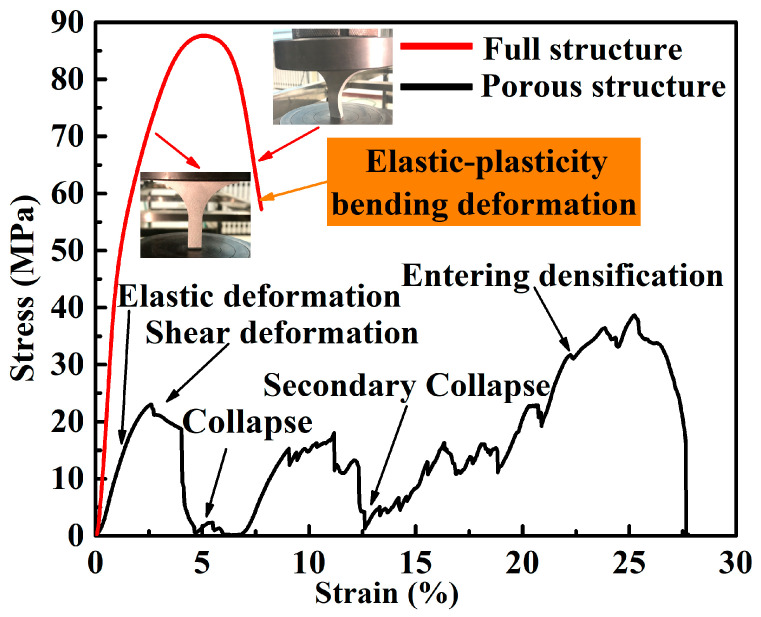
Compressive stress-strain curve of the tree column structures-type AlSi10Mg alloy branches.

**Figure 8:**
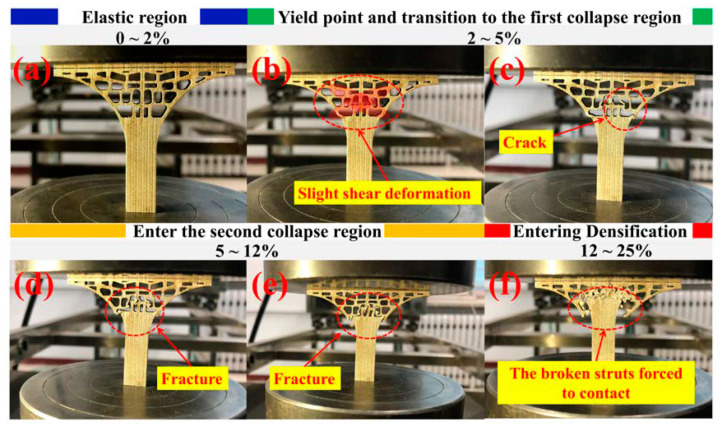
Deformation snapshots of the tree column structures-type AlSi10Mg alloy branches at different deformation stages under compression: (**a**) elastic region, (**b**,**c**) yield point and transition to the first collapse region, (**d**,**e**) the second collapse region, (**f**) densified region.

Figure 7 showed the stress-strain curve distribution of the tree column structures-type AlSi10Mg alloy branches and a full (non-porous) structure samples with an identical outline. Insets are photographs showing the elastic-plasticity bending deformation of a full (non-porous) structure samples with the identical outline, and the deformation mainly occurred in the support part of the lower half of the alloy sample. Further analysis showed that the compressive strength of the compact sample is poor, and the ultimate compressive strength (UCS) is obtained when the strain is 5.09%. In contrast, for the tree column structures-type AlSi10Mg alloy branch, the AlSi10Mg alloy branch showed relatively tortuous deformation behavior, with a strong compression resistance up to approximately 25.22% strain before entering densification (Table 2), indicating excellent ductility. Compared with the conventional AlSi10Mg lattice structures manufactured by SLM (the entire structure has a regular and evenly distributed pore structure, and it usually has four different deformation stages: elasticity, shear, collapse, and densification), the branches with the lower part of the supporting body and the upper part of the porous body in this experiment collapsed twice. This as based on the fact that the stress concentration at the interface (dense body and porous body) of AlSi10Mg alloy branches with tree column structure was relatively serious and collapsed first (Figure 8c). With the compression continued, the upper part of the porous body also began to collapse (Figure 8d,e). Finally, with the further compression, the upper rigid plate and the bottom rigid plate squeezed the tree column structures-type AlSi10Mg alloy branches, leading the porous branches to enter the densification stage, which made the broken debris or struts forced to contact (Figure 8f).

**Figure 9:**
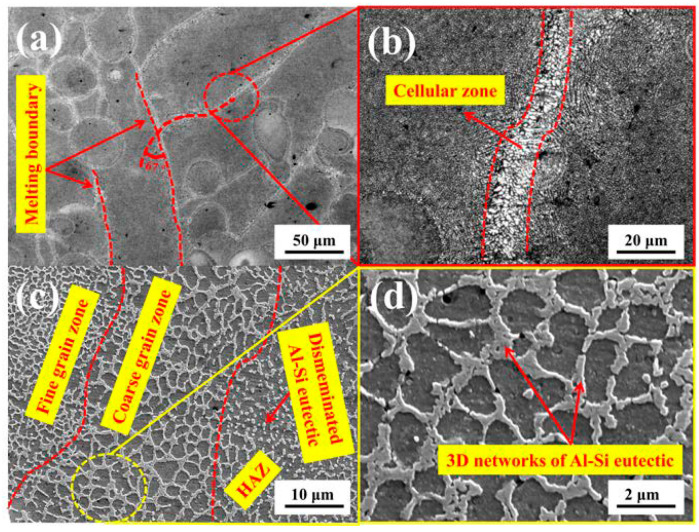
The microstructure of the tree column structures-type AlSi10Mg alloy branches on the cross-section: (**a**,**b**) Metallographic micrographs and (**c**,**d**) SEM micrographs.

**Figure 10:**
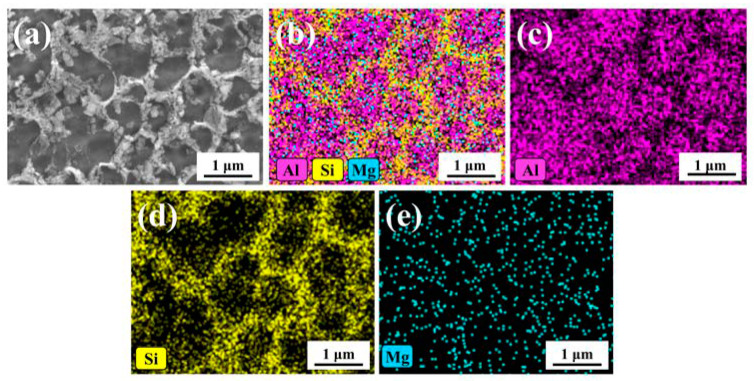
Energy-dispersive spectroscopy (EDS) analysis on the cross-section: (**a**) Morphology of cross-section, (**b**) Overall distribution diagram of elements, (**c**–**e**) The elemental distribution diagram of Al-Si–Mg on the cross-section.

Figure 9 showed the microstructure and local enlarged morphology of tree column structures-type AlSi10Mg alloy branches. Combined with the optical micrograph shown in Figure 9b, it can be seen that the dendrites at the molten pool boundary were coarser than the inside of the molten pool and grown in a cell (melting boundary, Figure 9a). Figure 9c,d showed the high-magnification SEM morphology and the local enlarged image of the branches. Figure 10 elucidated the energy-dispersive spectrometer (EDS) image of the tree column structures-type AlSi10Mg alloy branches on the cross-section. It can be seen that the main component of the 3D network distributed on the cross-section is Al-Si eutectic, while Al and Mg are relatively homogeneous. Furthermore, according to the density and morphology of the 3D networks of Al-Si eutectic, it can be divided into three typical regions: the fine-grained area located inside the molten pool, the heat-affected zone (HAZ) formed by the secondary heating of laser during the scanning process, and the coarse-grained area at the boundary of the molten pool. There were obvious differences in the morphology and size of Al-Si eutectic networks in the three regions. In the fine-grained area inside the molten pool, Al-Si eutectic precipitated between primary α-Al dendrites (at the eutectic temperature, the Si and Al phases form in between the primary dendrites), showing a fine network structure. However, the coarse grain zone was located at the boundary of the molten pool, and the temperature at the boundary of the molten pool was relatively lower compared with the temperature inside the molten pool (the primary dendrite size is a function of solidification rate—larger dendrites are a result of slower cooling rates). Furthermore, the primary aluminum dendrites are grown in the form of cellular crystals at the boundary of molten pool, which resulted in the sparse cellular morphology of the Al-Si eutectic structure. For the HAZ, due to the thermal cycle caused by laser secondary heating, the primary dendrites and Al-Si eutectic were reheated (likely beyond the solidus or liquidus and quickly cooled), such that a lot of Si was trapped in the primary Al. Therefore, the Al-Si eutectic network structure was seriously decomposed (Figure 9c).

**Figure 11:**
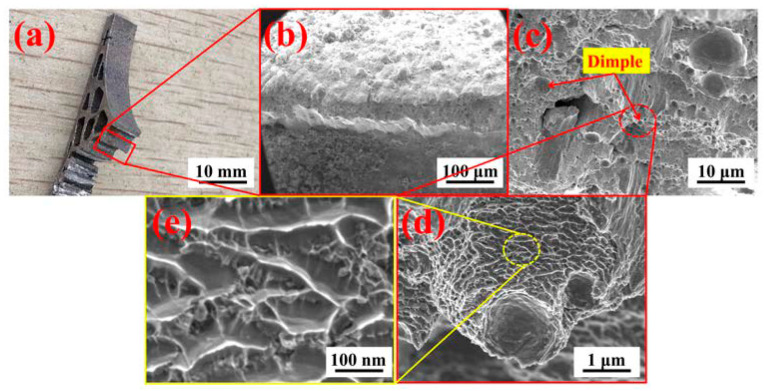
Photograph and SEM micrographs of fractured surfaces of the tree column structures-type AlSi10Mg alloy branches. (**a**) Photograph and (**b**–**e**) SEM micrographs.

Figure 11 showed the complete fracture mechanism of the tree column structures-type AlSi10Mg alloy branches and found that the fracture surfaces were accompanied by dimples with different sizes (the fracture mode was a ductile fracture, which was related to the 3D network morphology of the Al-Si eutectic). During the compression process, due to the low strength of the matrix α-Al phase, the plastic deformation first began with the α-Al phase. However, the 3D networks of the Al-Si eutectic surrounded the α-Al phase, and the Al-Si eutectic had high strength, resulting in stress concentration at the Al/Al-Si eutectic interface. When the stress level reached the ultimate strength of the two-phase interface, micropores were formed on the interface. As the compression continued, the micropores gathered and grew and eventually broke, resulting in the appearance of the dimples (Figure 11c–e). Furthermore, the 3D networks (Al-Si eutectic) of AlSi10Mg manufactured by SLM had three different morphologies (Figure 9c), and the different morphologies of the Al-Si eutectic network directly affected the formation position and quantity of micropores, and then it directly affected the morphology and size of fracture dimples (Figure 11c–e).

**Figure 12:**
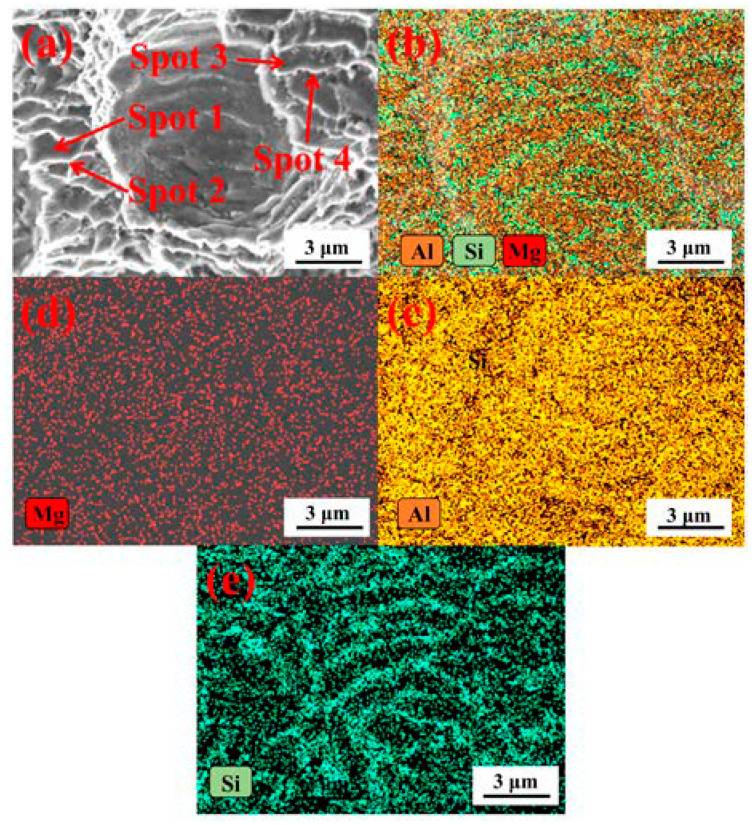
EDS analysis of fracture surface: (**a**) The fracture morphology, (**b**) Overall distribution diagram of fracture elements, (**c**–**e**) The elemental distribution diagram of Al-Mg-Si on the fracture surface.

Figure 12 showed the EDS analysis of the fracture surface. The Al element and Mg element of the tree column structures-type AlSi10Mg alloy branches were evenly distributed on the fracture surface, and the influence of the unevenness of the fracture surface on the distribution of fracture elements was excluded (Figure 12c,d). Moreover, the distribution of the Si element on the fracture surface was related to the distribution of the dimples (Figure 12e).

**Figure 13:**
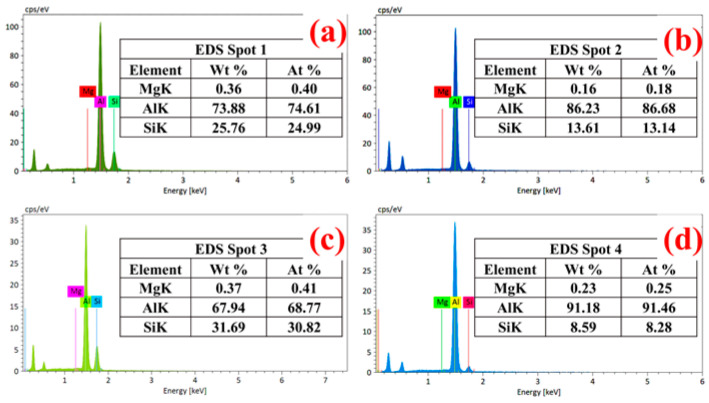
Spot analysis of fracture surface: (**a**,**b**) Spot 1, Spot 2, and (**c**,**d**) Spot 3, Spot 4.

In order to further quantitatively analyze the Si content in the dimple, the corresponding spot analysis was carried out on the bottom of the dimple and the edge of dimple (Figure 13). It was found that the Si content at the bottom of the dimple was 25.76 and 31.69 wt % (Spot 1, Spot 3 Figure 13a,c), while for the dimple edge, the Si content was only 13.61 and 8.59 wt % (Spot 2, Spot 4 Figure 13b,d). Therefore, a key conclusion was established: the Si content at the bottom of the dimple is higher than that at the edge of the dimple. Micropores gathered and grew until rupture occurred, which was the root cause of the Si content at the bottom of the dimple being higher than that at the edge of the dimple.

## 4. Conclusions

(1) When the tree column structures-type AlSi10Mg alloy branches were compressed along its building direction, the branches collapsed twice, which had typical elastic, shear, collapse, and densification stages.
(2) The interface between the supporting body and porous body of the tree column structures type AlSi10Mg alloy branches designed in this study was the concentrated position of stress and strain distribution, and the fracture at this position was prior to other positions.
(3) 3D networks of the Al-Si eutectic had three different morphologies, which affected the morphology and the size of dimples.
(4) The Si content in the dimple of the tree column structures type AlSi10Mg alloy branches prepared by SLM was higher than that in the dimple edges.

## Figures and Tables

**Table 1:** The main compositions of AlSi10Mg spherical powder.

Element	Al	Si	Mg
weight %	89.35	9.8	0.35

**Table 2:** The ultimate compressive strength (σ) and strain-to-failure (ε) of the tree column structures-type AlSi10Mg alloy branches and a full (non-porous) structure samples with identical outline.

Model	σ _UCS_ (MPa)	ε _UCS_ (%)
Full structure	87.64 ± 0.03	5.09 ± 0.10
Porous structure	38.65 ± 0.10	25.22 ± 0.02
